# Effects of Polymer Properties on Solid-State Shear Pulverization: Thermoplastic Processability and Nanofiller Dispersibility

**DOI:** 10.1021/acsapm.2c01932

**Published:** 2023-02-09

**Authors:** Tyler A. Will, Yiran Lu, Katsuyuki Wakabayashi

**Affiliations:** Department of Chemical Engineering, Bucknell University, Lewisburg, Pennsylvania 17837-2029, United States

**Keywords:** processing, solid-state shear pulverization, polyolefins, polymers, plastics

## Abstract

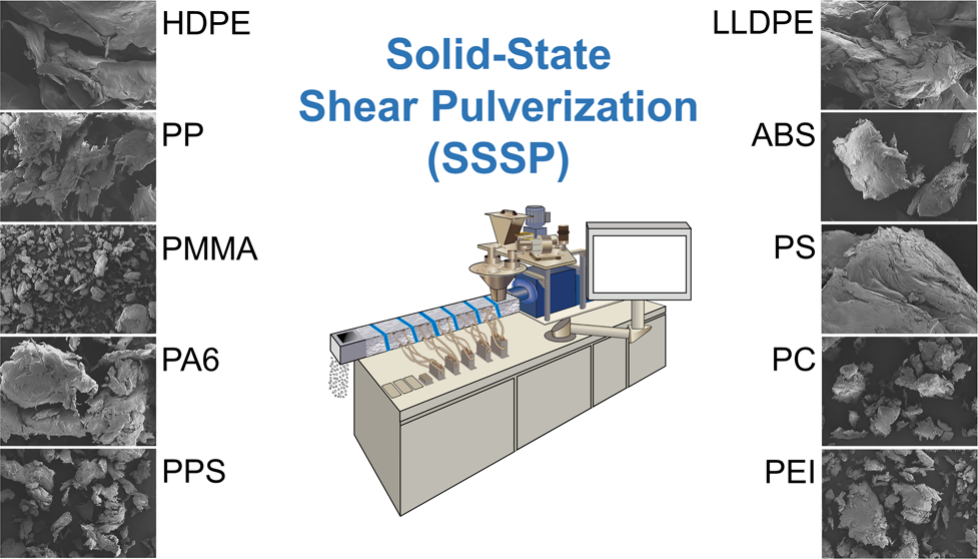

Solid-state shear pulverization (SSSP) is an alternative polymer processing technique based on twin-screw extrusion with a continuous cooling system. In SSSP, low-temperature mechanochemistry modifies the macromolecular architecture and morphology, which in turn leads to physical property changes in the material. While a wide range of homopolymers, polymer blends, and polymer (nano)composites have been previously developed with SSSP, a fundamental understanding of how mechanochemistry affects polymer chain architecture and structure, and in turn, material properties, has not been elucidated. This paper conducts a systematic processing–structure–property relationship investigation of 10 thermoplastic polymers with varying properties, as they are subjected to consistent SSSP mechanochemical pulverization and nanocomposite compounding. Structural, mechanical, and thermal characteristics of the neat polymers are correlated to their response to SSSP by way of process covariants. Further, we investigate how SSSP processing parameters cause structural changes such as molecular weight reduction and filler dispersion level, which in turn dictate system properties like melt viscosity and thermal stability. Mechanochemical engagement with a high degree of physical contact during pulverization and compounding, characterized by the SSSP covariants exhibiting specific mechanical energy values above 4 kJ/g and an average screw temperature above 20 °C, is ensured when polymers have a glass transition temperature below the processing temperature (<50 °C) and high toughness (>40 MPa). Crystallinity and low thermal diffusivity (<0.2 mm^2^/s) are additional factors for engaged SSSP processing. Chain scission is an unavoidable outcome of SSSP, though the associated molecular weight reduction was <10% for 7 out of 10 polymers. The elucidated processing–structure–property relationships would allow the SSSP process for a given polymer system to be tailored to the specific needs for molecular structure alterations and performance improvements.

## 1 Introduction

While thermoplastic materials continue to meet the global demand for the consumer market with well-established large-scale compounding, extrusion, and molding methodologies, society’s technical, economic, and sustainability goals lead to the development of a new generation of polymeric materials and products.^1^ Twin-screw extrusion (TSE) is one of the most prominent and versatile methodologies for polymer mixing, compounding, and transferring,^2−4^ which has evolved into advanced and modified polymer processing methods involving sonication,^5,6^ supercritical fluid injection,^7,8^ and high-speed rotation.^9^ Solid-state shear pulverization (SSSP) is one alternative TSE-based technique that can tailor the macro-, micro-, and nanoscale structures of polymeric materials to achieve specific properties and performance.^10−28^ High compressive and shearing forces are applied at low temperatures, which results in the repeated fragmentation and fusion of the material. The associated mechanochemistry leads to the modification of the macromolecular structure and promotes the intimate mixing and homogeneous dispersion of multicomponent systems. Continuous and commercially scalable SSSP is an environmentally conscious process because it does not require additional resources such as heat, solvents, monomers, or additives (stabilizers, chain extenders, or cross-linking agents). The SSSP technology has proven to be effective in the in situ compatibilization of immiscible polymer blends,^11−13^ dispersion of unmodified filler particles in composites and nanocomposites,^14−17^ development of sustainable polymeric materials,^18−20^ and even in initiating molecular changes to homopolymers.^21−23^

Previous investigations suggest that not all polymers respond to the compressive and shearing mechanism of SSSP in the same fashion. The most studied polymer matrix and majority phase are polyolefins; polyethylene (PE) and polypropylene (PP) have shown to engage well with additives, fillers, and blend components effectively, as evidenced by significant physical property improvements.^11−18,20,25−27^ However, there has not been a fundamental understanding of the physical and chemical characteristics of polyolefins that render them more favorable to SSSP processing compared to other polymer types. In this study, 10 different polymers selected among several commercial thermoplastic categories are subjected to identical SSSP processing conditions, and the process response and output materials are evaluated for a systematic comparison. Elucidating more comprehensive processing–structure–property relationships with a wider range of polymeric materials would lead to a broader understanding of the real-world applicability of SSSP.

In conventional melt extrusion, processing parameters such as barrel temperature and screw speed (i.e., shear rate) influence the viscoelastic behavior of the molten polymer, dictated by rheology along with thermodynamics and transport kinetics of the system. In contrast, SSSP occurs in the solid phase and therefore the associated processing mechanisms are different and more complex. The material’s fundamental physical characteristics, such as stiffness and toughness, are expected to directly influence the macroscopic deformation and damage. Thermal properties are also important as the shear heat generated from pulverizing solid polymers needs to be actively removed in maintaining a low processing temperature to keep the material in the solid state.^4,29^ The mechanochemical process results in not only macroscopic morphological changes like size reduction, but also permanent molecular changes, such as free-radical formation, chain scission, and branch formation.^21,22,26,28^

This 10-polymer comparison report is divided into two sections. In the first part, thermal, mechanical, and rheological properties of as-received neat polymers were evaluated and correlated to SSSP output and processing characteristics; polymers were individually processed, and identical pulverization parameters were used for each material. In the second part, a model nanocomposite containing natural graphite was SSSP-compounded with each polymer. Graphite was chosen because it is an effective high-aspect-ratio filler that can be exfoliated and dispersed as nanoplatelets in a tunable fashion by SSSP.^14,17^ The success levels of filler incorporation were compared via structural, thermal, and rheological property characterization of the nanocomposites. The two studies give insight into how different thermoplastic types and characteristics respond to SSSP processing.

## 2 Experimental Section

Ten thermoplastic polymers were processed under consistent SSSP conditions in two distinct investigations: the first is a neat polymer study where the polymer was pulverized independently, for evaluation of its response and engagement to SSSP processing; the second is a nanocomposite study where a graphite filler was compounded with the polymer, to understand the effect of process parameters on its structure and properties.

### 2.1 Materials

#### 2.1.1 Thermoplastic Polymers

Ten commercial thermoplastic polymers were chosen to represent major industrial plastic categories. In the semicrystalline commodity thermoplastics category, LyondellBasell Petrothene LR 7320 (ρ = 0.953 g/cm^3^; MFI = 0.30 g/10 min@190 °C, 2.16 kg) was chosen for high-density polyethylene (HDPE), Dow DFDA-7059 (ρ = 0.918 g/cm^3^; MFI = 2.0 g/10 min@190 °C, 2.16 kg) for linear low-density polyethylene (LLDPE), and Total Petrochemicals PP3276 (ρ = 0.905 g/cm^3^; MFI = 2.0 g/10 min@230 °C, 2.16 kg) for polypropylene (PP). In the amorphous commodity thermoplastics category, Sabic CYCOLAC MG38 (ρ = 1.05 g/cm^3^; MFI = 15 g/10 min@220 °C, 10 kg) was chosen for acrylonitrile butadiene styrene (ABS), Arkema Plexiglas V045 (ρ = 1.19 g/cm^3^; MFI = 2.3 g/10 min@230 °C, 3.8 kg) for poly(methyl methacrylate) (PMMA), and Americas Styrenics STYRON 685D (ρ = 1.04 g/cm^3^; MFI = 1.5 g/10 min@200 °C, 5.0 kg) for polystyrene (PS). In the engineering thermoplastics category, BASF Ultramid B27 (ρ = 1.13 g/cm^3^; MFI = 150 g/10 min@275 °C, 5 kg) was chosen for polyamide 6 (PA6) to represent the semicrystalline type and Covestro Makrolon 2405 (ρ = 1.2 g/cm^3^; MFI = 20 g/10 min@300 °C, 1.2 kg) was chosen for polycarbonate (PC), which is amorphous. In the last category of high-performance thermoplastics, Solvay Ryton QA200N (ρ = 1.34 g/cm^3^; MFI = 100 g/10 min@316 °C, 5.0 kg) was chosen for polyphenylene sulfide (PPS), which is semicrystalline, and Sabic ULTEM 1000 (ρ = 1.27 g/cm^3^; MFI = 9 g/10 min@337 °C, 6.6 kg) was chosen for polyetherimide (PEI), which is amorphous. Prior to any SSSP processing, the polymer pellets were dried in a Precision Scientific Model 19 vacuum oven according to the recommendations outlined in the manufacturers’ specification sheets.

#### 2.1.2 Graphite Filler

Natural graphite was chosen as the model filler material for the compounding study; graphite exists as flakes of microns in size, and the stacked sp^2^-hybridized graphene layers can be exfoliated and dispersed to varying degrees with SSSP.^14,17^ Grade 230U natural flake graphite (average particle size = 20 μm, density = 2.2 g/cm^3^) from Asbury Carbons was used as-received, without chemical, thermal, or expansion treatment.

### 2.2 Processing

**Figure 1:**
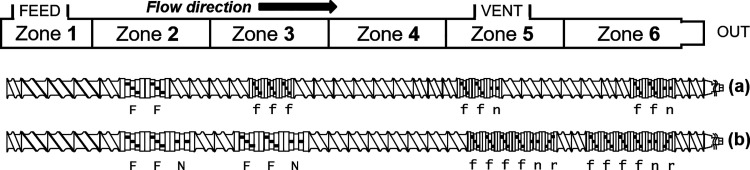
Schematic of the SSSP screw configurations employed in this study. (a) Moderate design contains 11 pulverization elements and (b) harsh design has 18. Letters in the diagram denote the types of pulverization elements. Capital letters are large kneading discs (L/D = 1.25) and lowercase small kneading discs (L/D = 0.75); f = forward-driving, n = neutral, r = reverse-driving.

Solid-state shear pulverization was performed using a modified KraussMaffei Berstorff ZE25-UTX twin-screw extruder with a diameter of 25 mm and a L/D ratio of 35. Low barrel temperatures were achieved with a continuous flow of −12 °C ethylene glycol/water coolant provided by a Budzar Industries BWA-AC-10 chiller. Figure 1 displays the screw element configurations. The neat polymer study employed a moderate configuration with forward and neutral kneading elements spanning a total of 9.25 out of 35 L/D in length. The nanocomposite production used a harsh configuration to properly exfoliate the graphite layered structure; forward, neutral, and reverse kneading elements were placed along 17.25 out of 35 L/D. A constant screw rotation speed of 200 rpm was used in both studies.^27^

Polymer pellets were metered into the Zone 1 hopper using a Brabender Technologie Model DS28-8 loss-in-weight feeder. The throughput of base polymer was kept constant at 300 g/h. For the first neat polymer processing study, the polymer passed through the SSSP instrument once. In the second filler compounding study, graphite filler at the loading level of 2 vol % was also fed into the Zone 1 hopper by a Brabender Technologies DDSR12-1 gravimetric powder feeder, and the combined materials were processed one, three, and five times (1, 3, and 5 passes, respectively) to compare the degree of filler exfoliation.^17^ The SSSP screw temperature was measured using an Omega OS102E IR temperature sensor, and the power consumption of the extruder motor was monitored by an Autonics LA8N watt-hour meter. Energy expenditure was calculated by integrating the power usage over the steady-state processing period.

Separately, control samples of each polymer with 2 vol % graphite nanocomposites were prepared via conventional melt mixing to serve as a reference for structural characterization. An Atlas Laboratory cup-and-rotor mixer was used to compound 3 g batches with the rotor speed of 170 rpm at a temperature that is 40–50 °C above the melt temperature (*T*_m_) or glass transition temperature (*T*_g_) of the respective base polymer. The SSSP and batch melt mix output materials were further processed for physical property characterization. Flat sheets ∼1 mm thick were compression-molded using a Carver Model C Press. A pressure of 700 kPa was applied at an isothermal temperature, 20–30 °C above *T*_m_ or *T*_g_ of the base polymer. Each specimen was subsequently air-cooled to room temperature and stored for at least 24 h prior to testing.

### 2.3 Characterization

Static uniaxial tensile testing of the neat polymers was performed based on ASTM D1708, using a Test Resources 313-47 Universal Testing Machine with an S2000N load cell and Newton control software. The cross-head speed of 10 mm/min corresponded to a strain rate of 0.01 s^–1^. Dynamic mechanical analysis (DMA) was performed on TA Instruments RSA 3 in tension mode. A temperature ramp was programmed from −100 °C to a temperature 30 °C above the polymer’s *T*_m_ or *T*_g_, at 5 °C/min with an amplitude of 0.1% and frequency of 1 Hz.

Specific heat capacity (*C*_p_) measurement was conducted on a TA Instruments Q2000 differential scanning calorimeter (DSC), calibrated with indium and sapphire standards. A modulated DSC method was programmed with a ramp rate of 2.0 °C/min at an amplitude of ±1.0 °C every 2 min over the temperature range of −10 to 110 °C,^30^ and the reversible *C*_p_ values were recorded. Thermal diffusivity (α) was measured at Cornell Energy Systems Institute, using a Linseis laser flash analyzer XFA 500. Compression-molded discs with a diameter of 25.4 mm were measured in 10 s intervals at ambient temperature.

Structural characterization of the SSSP-processed materials was conducted with a Hitachi SU 5000 FE scanning electron microscope (SEM) equipped with a secondary electron detector, using an accelerating voltage of 3.0 kV. The imaging surface was sputter-coated with gold using a Denton Vacuum Desk V. X-ray diffraction (XRD) of polymer/graphite nanocomposite samples was conducted on a PANalytical X’Pert Pro Multi-Purpose Diffractometer with Cu Kα monochromatic rays at 45 kV and 40 mA. The goniometer detector was set to scan between 2θ = 8° and 40° at 0.2° increments.

Thermogravimetric analysis (TGA) was performed on composite samples to verify the graphite content and measure the thermal degradation temperature in a nitrogen environment. TGA was performed on a TA Instruments SDT-Q600, calibrated with indium and tin, in a 10 °C/min heating ramp mode. The temperature at which a 5 wt % loss of the specimen occurred was recorded as the thermal degradation temperature (*T*_deg_).

Melt rheology was conducted on both neat and processed polymers using a TA Instruments DHR-2 with 25 mm parallel plate geometry in a nitrogen environment. For zero-shear viscosity determination, a steady-state shear flow sweep mode was employed with shear rates ranging from 0.001 to 100 s^–1^. For polymer nanocomposite morphology characterization, an oscillatory frequency sweep mode was programmed from 100 to 0.001 s^–1^ at 0.1% strain, while an appropriate testing temperature was selected for each polymer sample, typically within 20 °C of its MFI measurement temperature as outlined in the manufacturer’s specification sheet.

## 3 Preliminary Results: As-Received, Unprocessed Polymer Properties

The manner in which different polymeric materials respond to solid-state, low-temperature shearing and kneading action in SSSP processing is likely dictated by their inherent neat mechanical and thermal behavior. Therefore, we first established the baseline fundamental properties of the 10 polymers, as they were received. Table 1 summarizes the basic thermomechanical properties that we measured and analyzed in-house. The *T*_g_ and *T*_m_ (if semicrystalline) values were determined by the tan δ peaks in DMA. Two thermal transport quantities were measured at room temperature; *C*_p_ is expressed on a unit volume basis because extruder-based processing depends on factors such as screw filling ratio and surface contact for its heat transfer phenomena, and we included α to encompass the overall heat dissipation tendencies of the polymers. For mechanical properties, Young’s modulus (*E*), yield strength (σ_*y*_), strain at break (ϵ_b_), and tensile toughness (*U*_T_) were compiled from room-temperature tensile testing.

**Table 1:** Thermal and Mechanical Properties of Unprocessed, As-Received Polymer Samples

	*T*_g_	*T*_m_	*C*_p_@25 °C	α@25 °C	*E*@25 °C	σ_*y*_@25 °C	ϵ_b_@25 °C	*U*_T_@25 °C
polymer	(°C)	(°C)	(J/cm^3^-K)	(mm^2^/s)	(GPa)	(MPa)	(%)	(MPa)
HDPE	–110	121	1.7	0.32	1.2 ± 0.04	29 ± 1	630 ± 30	110 ± 8
LLDPE	–20	113	2.0	0.19	0.3 ± 0.02	12 ± 1	950 ± 60	159 ± 18
PP	–1	150	1.5	0.15	1.3 ± 0.02	37 ± 1	560 ± 110	124 ± 28
ABS	–82, 110		1.3	0.12	1.7 ± 0.04	47 ± 1	24 ± 7	10 ± 3
PMMA	108		1.6	0.12	2.7 ± 0.13	74 ± 4	3.4 ± 0.4	1.4 ± 0.2
PS	109		1.2	0.14	2.0 ± 0.06	42 ± 3	4.4 ± 0.4	0.9 ± 0.1
PA6	47	210	1.6	0.16	2.2 ± 0.12	81 ± 7	65 ± 14	43 ± 16
PC	142		1.4	0.14	1.8 ± 0.03	74 ± 1	6.5 ± 0.6	3.8 ± 0.8
PPS	110	277	1.2	0.15	2.9 ± 0.04	99 ± 3	4.8 ± 0.3	2.8 ± 0.3
PEI	221		1.3	0.12	2.5 ± 0.11	112 ± 12	6.6 ± 1.2	4.5 ± 1.3

While the values reported in Table 1 are typical for respective commercial-grade thermoplastics, a comparison across the different types highlights notable differences. In particular, *T*_g_ varies over a 330 °C span, and *T*_m_ values differ by as large as 160 °C. For mechanical properties, the ductility and toughness differences are over 280- and 175-fold, respectively, between the lowest and highest values. These disparate tensile behaviors can be categorized into brittle, glassy polymers with little or no plastic deformation before fracture, versus ductile semicrystalline polymers with the ability to absorb significant mechanical energy before fracture.

**Figure 2:**
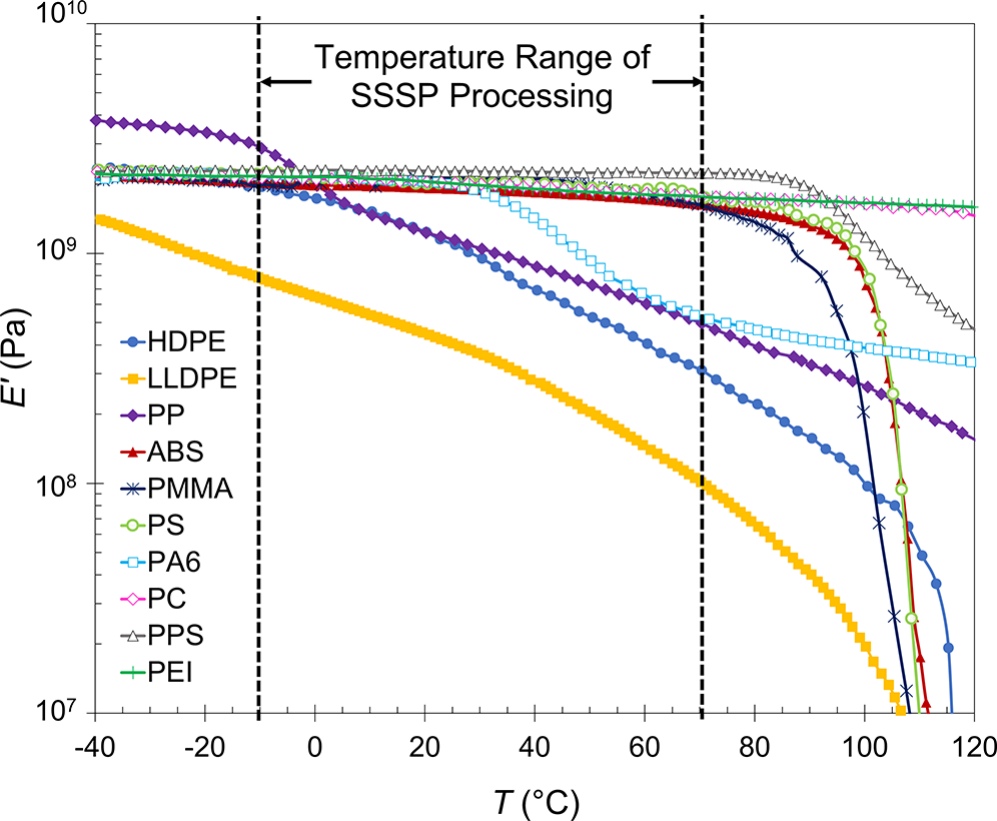
Storage modulus as a function of temperature for the 10 neat polymer systems, measured via dynamic mechanical analysis in tension mode.

While the temperature dependence of stiffness, strength, ductility, and toughness in these polymers is an important factor in their respective response to low-temperature SSSP processing, room-temperature tensile testing results in Table 1 are justified as a realistic and reasonable starting point to compare the 10 polymers, as 25 °C is approximately the median of the steady-state SSSP operation temperatures (∼−10 to 70 °C) of most polymeric materials. To probe the temperature dependence, Figure 2 compares the DMA storage modulus (*E*′) curves for this relevant temperature range. Six of the 10 polymers—ABS, PMMA, PS, PC, PPS, and PEI—have consistent high modulus values on the order of 2–3 GPa throughout the temperature range of interest, while almost all of the semicrystalline polymers in the group—HDPE, LLDPE, PP, and PA6—exhibit a notable drop in elastic modulus with increasing temperature; PP and PA6 even experience devitrification, depicted by the step changes in *E*′ vs *T*. The only exception to this trend was PPS; even though PPS is a semicrystalline material, its *T*_g_ is higher than the evaluated temperature range; therefore, its behavior was more similar to a glassy material, with a steady *E*′ plateau in the region.

## 4 Results and Discussion

### 4.1 SSSP Processing of Neat Polymers

We proceed with the first study, where 10 neat thermoplastic materials are individually subjected to an identical SSSP process. The applied set of parameters, i.e., screw design in Figure 1(a), 200 rpm speed, 300 g/h throughput, and −12 °C chiller setting, were chosen to accommodate the differing responses from 10 polymers, and attempt to maintain solid-state processing; these processing conditions constitute one of the milder runs that have been used in prior SSSP work.^22^

#### 4.1.1 Output Appearance

All materials were received in the form of commercial pellets, ∼3 to 5 mm in length, and thus size reduction is one of the first expected outcomes of solid-state processing, though physical deformation, chemical reaction, heat dissipation, and change in micro/nanostructure are some of the other possible outcomes of comminution of polymers.^31,32^ In this section, discussions on particle deformation and size reduction are limited to observations with single-pass SSSP runs. A full understanding of the deformation mechanism in SSSP would result from detailed fractional and multiple pass analysis on an individual polymer basis.^28^

**Figure 3:**
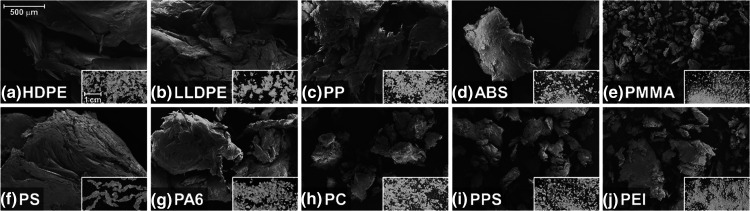
Scanning electron micrographs of SSSP-processed neat (a) HDPE, (b) LLDPE, (c) PP, (d) ABS, (e) PMMA, (f) PS, (g) PA6, (h) PC, (i) PPS, and (j) PEI. Insets are photographs of the direct SSSP output. The scale bar of 500 μm for the SEM and 1 cm for the inset photographs are applicable to all images in the set.

The SEM and photographic images of the SSSP output are compiled in Figure 3, all taken under equal magnifications. The particle size and shape, as well as the texture of the pulverized polymer surface, differ considerably. The size reduction responses of the 10 polymers can be classified into three general groups. First, isometric powder particles can be seen in ABS, PMMA, PC, PPS, and PEI. The relative sizes are drastically different; ABS and PEI contain relatively larger powder particles on the order of millimeters, while PMMA has one of the finest powder outputs in this study, around tens to hundreds of microns. One common fact of these powder output-producing samples is that they are glassy, brittle materials at ambient temperature because their *T*_g_ is well above the processing temperature, regardless of crystallinity. According to Table 1, the ambient temperature toughness of these five polymers is indeed relatively low at ≤10 MPa, which corroborates the physical, macroscopic behavior of the samples under mechanical compression.

The second commonly observed output response is a flat flake output, found in HDPE, LLDPE, PP, and PA6. The flakes are consistently on the order of millimeters in size, and micron-scale particles are rarely observed in these materials under the mild screw configuration used. The four thermoplastics have the highest ductility and toughness in Table 1, and are the same four identified as the only polymers that exhibit stiffness changes with a temperature ramp in Figure 2. In addition, the four are semicrystalline thermoplastics, whose *T*_m_ values are well above, and *T*_g_ values around or below, the typical SSSP-processing temperature range. These thermomechanical characteristics commonly observed in the four polymers are responsible for the flake morphology development under SSSP. The ductile regime facilitates the mechanisms of plastic deformation and extensional shear, as well as fragmentation and fusion.^23,25^

Lastly, PS responded to the SSSP conditions in this study in an unexpected manner, resulting in a category of its own. The inset image of Figure 3(f) depicts a strand segment output, rather than flake or powder output observed in other SSSP-processed materials. The smooth curvatures on the edges of the corresponding SEM image suggest that the polymer had devitrified and subsequently revitrified in the barrels during the SSSP process. In prior experience, PS has been SSSP-processed into fine powder, akin to PMMA and other brittle polymers above, when the throughput is lower than 300 g/h or when the screw design is set even milder than the mild screw configuration employed in this study (Figure 1(a)). Therefore, PS undergoing SSSP has a distinct tendency to increase in temperature and subsequently devitrify and soften, instead of being pulverized. The reason for this outlier behavior is not obvious, especially because our earlier analysis (Figure 2 and Table 1) indicated that the temperature-dependent stiffness trend of PS is essentially identical to that of PMMA, another amorphous thermoplastic sample with a single *T*_g_ around 110 °C; in fact, under normal characterization conditions, PS would devitrify at a slightly higher temperature than PMMA. A possible explanation is the fact that the PS specimen has the lowest *C*_p_ and one of the lowest α values compared to other polymers in the study. The frictional heat from the SSSP shear and compression is not easily dissipated from the polymer and raises its temperature toward its *T*_g_ more readily than other materials under the same shearing conditions. Further, comparing the tensile testing results between the PS and PMMA samples, PMMA is slightly more brittle, which may contribute to PMMA fracturing more readily into finer particles with very little frictional heat generation upon SSSP processing.

SSSP runs experiencing partial devitrification or melting are usually deemed irrelevant and out of scope for investigations like the current one, as they deviate from the mechanism of *solid-state* deformation and instead resemble flow deformation found in conventional melt extrusion. However, in this paper, we continue to include PS in the comparison, with an acknowledgment that it was exposed to drastically different thermal, mechanical, and rheological conditions compared to the other nine samples.

#### 4.1.2 SSSP Process Covariants

**Figure 4:**
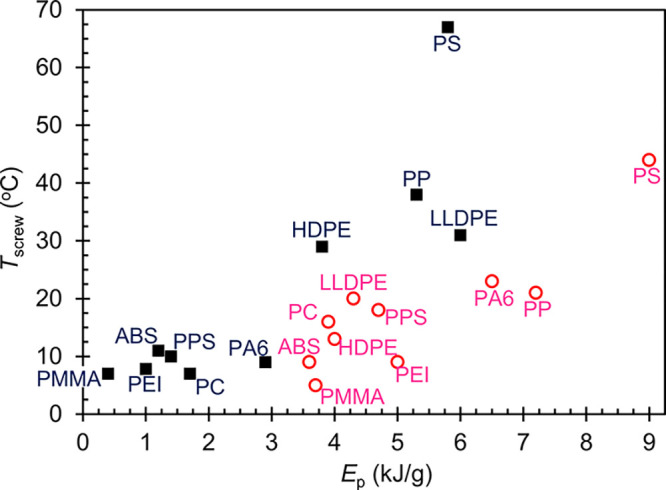
Process covariants, *T*_screw_ vs *E*_p_, from the neat polymer processing study (open circles) and the 1-pass polymer/graphite compounding study (filled squares).

Having established how the 10 polymers responded to the SSSP process under the same set of conditions and resulted in different size reduction and deformation mechanisms, we now evaluate the reciprocal, namely, how the SSSP instrument responded to the process of pulverizing each polymer. Two quantitative SSSP covariants are used. First, specific mechanical energy (*E*_p_) is the measure of mechanical energy expended per unit mass of sample by the motor; it is the simplest and most widely used metric for mechanical shear and compression.^23,27^ Second, average screw temperature (*T*_screw_) probes the general state of the thermal equilibrium between heat generated by the pulverizing sample and the heat removed by the cooling system. It is important to note, however, the recorded *T*_screw_ value is considerably lower, on the order of tens of degrees centigrade, than the actual temperature of the polymer being processed because (1) *T*_screw_ is a single average representation of a temperature profile throughout the length of the screw and (2) the SSSP screws are running only partially filled, resulting in parts of the screw that do not contain any material at any given time. The two covariants are plotted against each other in Figure 4 (open circles), where a general trend between *E*_p_ and *T*_screw_ is apparent; a polymer that consumes a relatively large mechanical energy for pulverization also tends to exhibit a relatively high equilibrium temperature. The variation of *T*_screw_ values about an implicit linear relationship in Figure 4 is due to the differences in *C*_p_ and α of individual polymers, as discussed earlier.

To relate this covariant information to the SSSP output appearance as well as the neat polymer physical properties from the earlier discussion, we focus on the select samples that generated significantly high and low *E*_p_ or *T*_screw_ values. First, PS has already been established as an anomalous sample, having partially devitrified in SSSP. A temperature above its *T*_g_ has been reached at some point along the SSSP screws, which is manifested by the highest recorded *T*_screw_ of the series. Similarly, the highest *E*_p_ was observed not only because of the higher torque required to process rubbery amorphous PS, but also because the devitrified and viscous PS material sticks to the partially filled screws and is retained in the barrels, raising its residence time and increasing its contact time with the rotating screws. The next highest *E*_p_ and *T*_screw_ are recorded in PP and PA6, which are semicrystalline, relatively stiff and tough thermoplastics, with flakey SSSP output according to Figure 3. The processing temperature was above the *T*_g_, but well below the *T*_m_. The high *E*_p_ values suggest that these polymers interacted with the kneading discs of the SSSP screws effectively with complete contact while deforming plastically and producing flat flake output. As a result of the shearing and compression, *T*_screw_ values increased but not to the point of melting the polymer in the SSSP instrument.

The lowest three *E*_p_ and *T*_screw_ values come from the amorphous polymers of the series (other than PS), which commonly produced powder output according to Figure 3. Because the processing temperatures were well below the *T*_g_ values, the glassy polymers underwent brittle fracture when in contact with the kneading discs of the screws. As indicated by the relatively low *U*_T_ reported for these polymers, brittle fragmentation of the polymers to a powder, with sizes as low as ∼10 μm, does not take significant mechanical energy input. This observation is significant as it contradicts the findings of prior solid-state processing work where considerable mechanical energy was required to achieve significant size reduction of particles in commingled plastics and rubber recycling.^24,25,33^ While low *E*_p_ values may be favorable from an energy cost standpoint, they reflect the very low mechanochemical engagement and interaction opportunities that the glassy amorphous polymers have in an SSSP process; their negative implications on material development and compounding will be discussed later.

#### 4.1.3 Molecular Structure Changes

**Figure 5:**
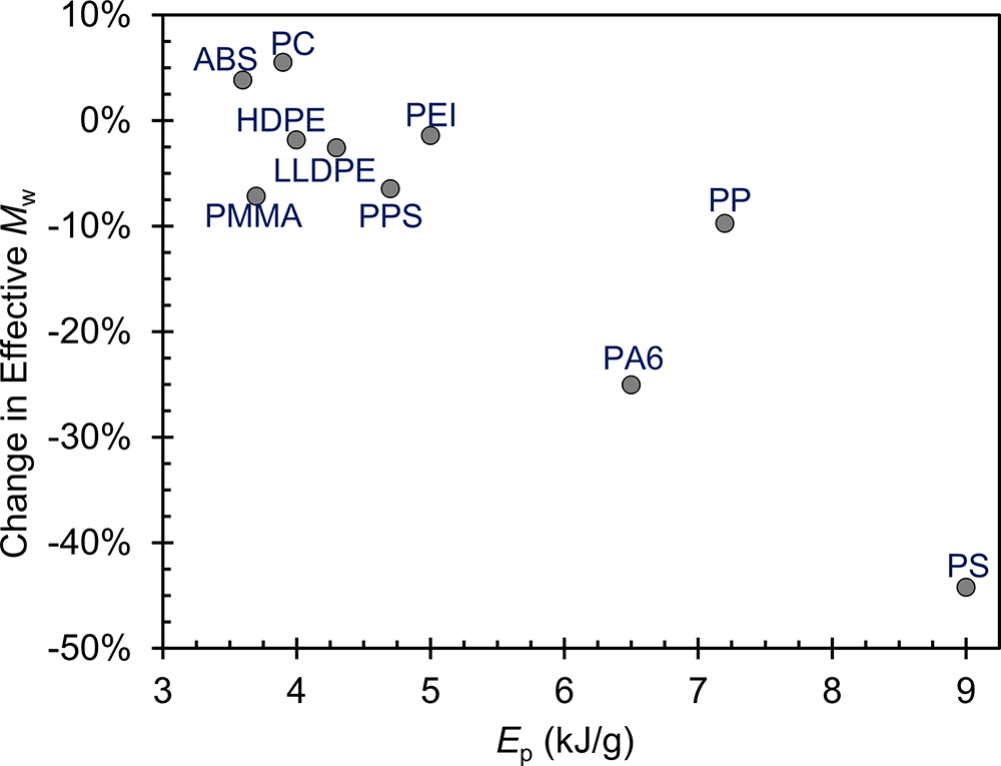
Change in effective *M*_w_ from SSSP, plotted as a function of *E*_p_.

Shear flow melt rheology was conducted on the series of samples before and after SSSP processing to determine the relative changes in the macromolecular structure. While melt viscosity is affected by both molecular weight and molecular weight distribution, we focused on evaluating the zero-shear viscosity (*η*_0_) difference between the initial pre-SSSP and post-SSSP samples to probe the primary effect of change in molecular weight (Table S1). Based on the established relationship between η_0_ and weight-average molecular weight (*M*_w_)^34,35^
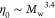
1we calculated the ratio of the η_0_ values, post- to pre-SSSP, raised to the reciprocal power to capture the linearized change in an effective *M*_w_ in each polymer. Different degrees of molecular degradations were observed, and a general correlation between effective *M*_w_ reduction and *E*_p_ expended by the polymer in its SSSP run is apparent, as seen in Figure 5. While we refrain from overanalyzing the results, we speculate that the molecular weight reduction corresponds to the level of physical engagement experienced by the polymer with the screws during the SSSP run.

Chain scission is a prominent aspect of mechanochemistry manifested in SSSP. Regarding the extent of chain scission, most polymers (all other than ABS, PC, and PS) exhibited levels of *M*_w_ reduction that are within an expected range.^22,23,25^ The PS sample suffered the most severe molecular weight degradation to an extent that neither conventional melt extrusion nor fully solid-state SSSP of commercial PS would yield. We speculate this 44% reduction in effective *M*_w_ is because of the partial devitrification occurring in the SSSP run, resulting in a slower starved flow of the material in the instrument, as discussed above. Excess chain scission occurred because SSSP conditions were not properly tuned to keep the polymer in the solid state.

In contrast, ABS and PC exhibited an apparent slight increase in η_*0*_ and thus in effective *M*_w_. The increase in viscosity upon SSSP processing, in which chain scission is essentially inevitable, points to potential chain branching as an additional phenomenon occurring in SSSP mechanochemistry. Prior work has also reported on chain branching in SSSP-processed homopolymers.^21,22,25^ Although the observed levels were minimal in the cases of ABS and PC, such molecular architecture changes as branching can yield a beneficial outcome for applications where higher toughness and impact resistance are needed.^36,37^ A complete understanding of the effects of SSSP-processing conditions on free-radical formation, leading to linear chain scission vs branching mechanisms, would be useful to conduct as future work.

It is well-established that SSSP processing of homopolymers, especially when using a mild screw configuration as used in this study, does not significantly alter their fundamental bulk thermal and mechanical properties such as devitrification and melting temperature, stiffness, and strength.^21,23,25^ Therefore, there is little value in conducting any thermomechanical characterization of the SSSP-processed homopolymers in this study. The next study incorporates a graphite additive using a harsher SSSP screw configuration. Pronounced differences in morphology and physical property modifications are expected across the 10 base polymers, and they are rigorously compared and correlated with the levels of engagement that the polymers exhibit with SSSP processing.

### 4.2 Polymer/Graphite Nanocomposite Study

Incorporating additives and fillers into a polymer matrix via a compounding technique is a common practice with industrial and value-add implications.^38^ For our model compounding study, natural graphite, rather than pre-exfoliated or thermally expanded analogues,^39,40^ was chosen as the filler,^14,17^ as its typically poor dispersion in polymer matrices allows the results among the 10 polymers to be differentiated more effectively. As shown in Table 2, we compounded graphite with each of the 10 polymers with a consistent filler content at 2 vol %. The natural graphite would undergo a transformation to graphite nanoplatelets if successfully exfoliated and dispersed in a polymer matrix.^14,41^

**Table 2:** Polymer/Graphite Nanocomposite Samples: Filler Content from TGA and STE from Melt Rheology

			STE, average				
	filler content, average						
	(1-, 3-, and 5-pass samples)			neat polymer	graphite nanocomposites		
base polymer	wt %	vol %	test temperature (°C)	as received	1-pass	3-pass	5-pass
HDPE	4.4 ± 0.2	2.0 ± 0.1	200	0.40	0.43	0.43	0.43
LLDPE	4.6 ± 0.2	2.0 ± 0.1	200	0.036	0.039	0.043	0.045
PP	4.7 ± 0.2	2.0 ± 0.1	210	0.11	0.18	0.33	0.37
ABS	3.9 ± 0.1	1.9 ± 0.1	230	0.84	0.85	0.86	0.86
PMMA	3.5 ± 0.1	1.9 ± 0.1	230	0.080	0.078	0.064	0.050
PS	4.1 ± 0.1	2.0 ± 0.0	200	0.022	0.050	0.76	0.86
PA6	3.8 ± 0.2	2.0 ± 0.1	240	0.066	0.39	0.49	0.52
PC	3.4 ± 0.2	1.9 ± 0.1	220	0.047	0.19	0.13	0.10
PPS	3.1 ± 0.1	1.9 ± 0.1	300	0.055	0.34	0.41	0.43
PEI	3.4 ± 0.1	2.0 ± 0.1	290	0.092	0.11	0.10	0.10

#### 4.2.1 Compounding Covariants

The 10 polymers were subjected to a new, identical set of processing conditions in the compounding study with 2 vol % graphite loading. A harsh screw configuration, shown in Figure 1(b), was employed, and furthermore, samples were made with three different SSSP-processing levels by way of 1, 3, and 5 passes.^17^ The graphite inclusion enabled the use of a harsher screw configuration, as graphite acts as a natural lubricant and suppresses the frictional heat from the continuous shearing and compression of the materials. The *E*_p_ and *T*_screw_ covariants from the 1-pass compounding runs are mapped in Figure 4 (filled squares), alongside the equivalent points from the earlier neat polymer processing study. The lubrication effect of the graphite is immediately apparent when comparing the *E*_p_ values involved in the present graphite study, involving a harsh screw configuration, to those in the neat processing study, using a mild screw configuration.^14,17^ Comparing the 10 graphite study data points in Figure 4, low *E*_p_ and *T*_screw_ were recorded in ABS, PMMA, PC, PPS, and PEI; those are the thermoplastics whose *T*_g_ are well above the processing temperatures. Soon after entering the SSSP instrument, the glassy polymer pellets underwent a brittle fracture into fine pieces, which prevented them from fully impinging the filler particles, even with a harsh screw configuration. Effective compounding with SSSP requires physical interfacial shearing and compression between the components, but these glassy polymers are evidently deprived of the necessary contacts due to premature self-size reduction along with graphite lubrication. In contrast, polyolefins (HDPE, LLDPE, and PP), with their semicrystalline and ductile nature, engage with graphite compounding very well, as higher *E*_p_ and *T*_screw_ values indicate. Lastly, PS retains relatively high *E*_p_ and *T*_screw_ in its graphite compounding run, as it is the only system that is processed in a partially devitrified state; longer residence time and the transition through the devitrification point resulted in an unusually high mechanical load and processing temperature.

#### 4.2.2 Filler Distribution and Exfoliation

**Figure 6:**
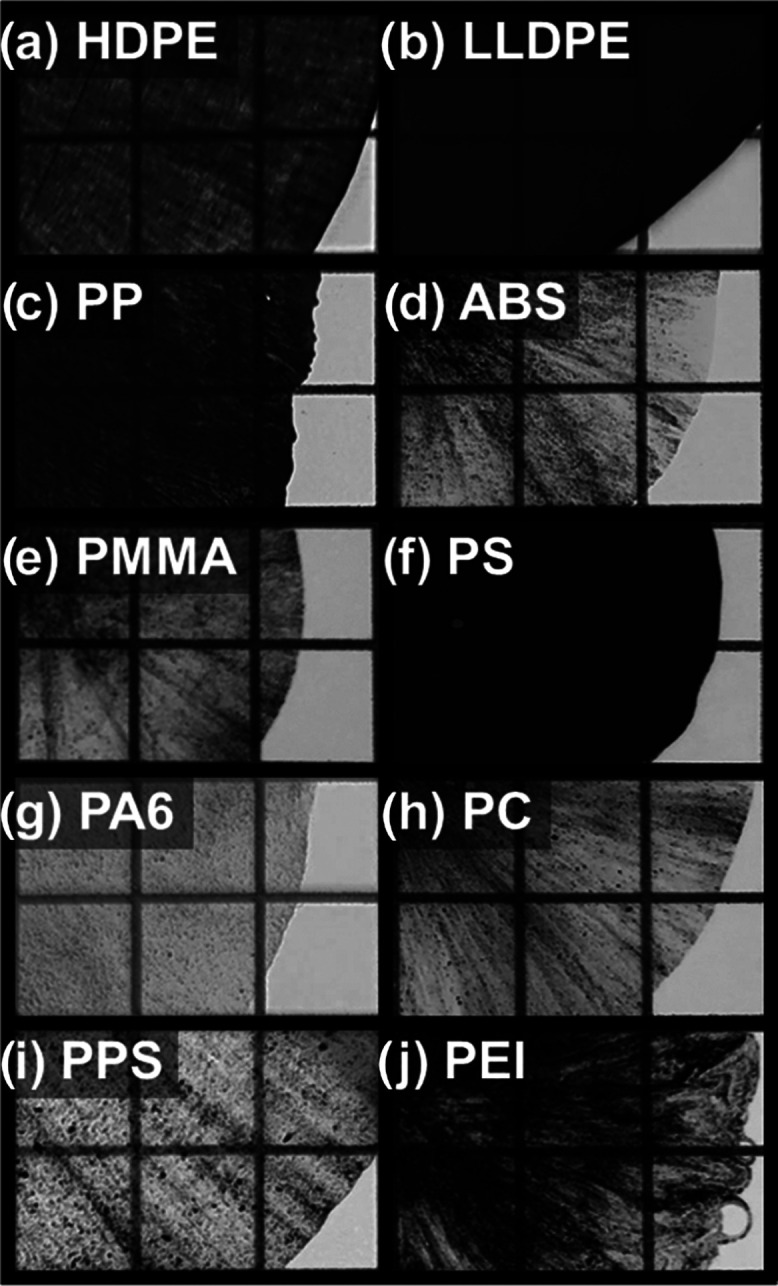
Melt-pressed thin-film specimens of 1-pass, 2 vol % graphite nanocomposites based on (a) HDPE, (b) LLDPE, (c) PP, (d) ABS, (e) PMMA, (f) PS, (g) PA6, (h) PC, (i) PPS, and (j) PEI. The photographs were taken against a light box background, and grids under the film are 5 mm × 5 mm.

The output of SSSP-processed nanocomposite runs closely resembled the corresponding neat processing samples in terms of the characteristic shape, size, and form (flakes vs powder vs strands). The powdery output of ABS, PMMA, PC, PPS, and PEI also accompanied distinct fine graphite particulates, indicating that not all of the graphite was homogeneously mixed in the polymer matrix. On the other hand, HDPE, LLDPE, PP, PA6, and PS each yielded a single uniform output with the graphite filler physically infused into the base polymer. To further illustrate the degree of graphite incorporation and distribution,^14^ the 1-pass SSSP-processed flakes and powders were melt-pressed into 0.05-mm-thick films. The visual appearance of the films was compared using a gridded light box, as seen in Figure 6. Graphite was more homogeneously distributed in PS and the three polyolefins (HDPE, LLDPE, and PP), whereas other samples showed disparate areas of missing filler and graphite streaks along the melt press flow pattern. The homogeneous distribution observed in the select samples can be tied to their higher *E*_p_ values recorded in Figure 4; higher material contacts with full compression and shearing corresponded to the graphite filler being more uniformly distributed throughout the polymer. Note that this comparison was for the 1-pass samples where the relative contrast among the 10 polymer systems was the greatest. For a given polymer base, subsequent 3-pass and 5-pass runs distributed the graphite more homogeneously, and the equivalent pressed films would appear markedly more uniformly black and opaque.

The high level of graphite distribution in the film of PS/graphite lends itself to a hypothesis that the interfacial interaction is based on the similar chemical structure between the benzene side groups of PS and sp^2^-hybridized carbon layers of graphite. Neither our previous nor current work has affirmed any favorable structural interaction. Instead, we believe that the homogeneous mixing of the filler was merely facilitated by compounding in both the solid and partially devitrified states. Previous studies have reported a similar finding, concluding that melt compounding immediately after solid-state processing led to a higher filler distribution than solid-state processing alone.^16,17,42^

**Figure 7:**
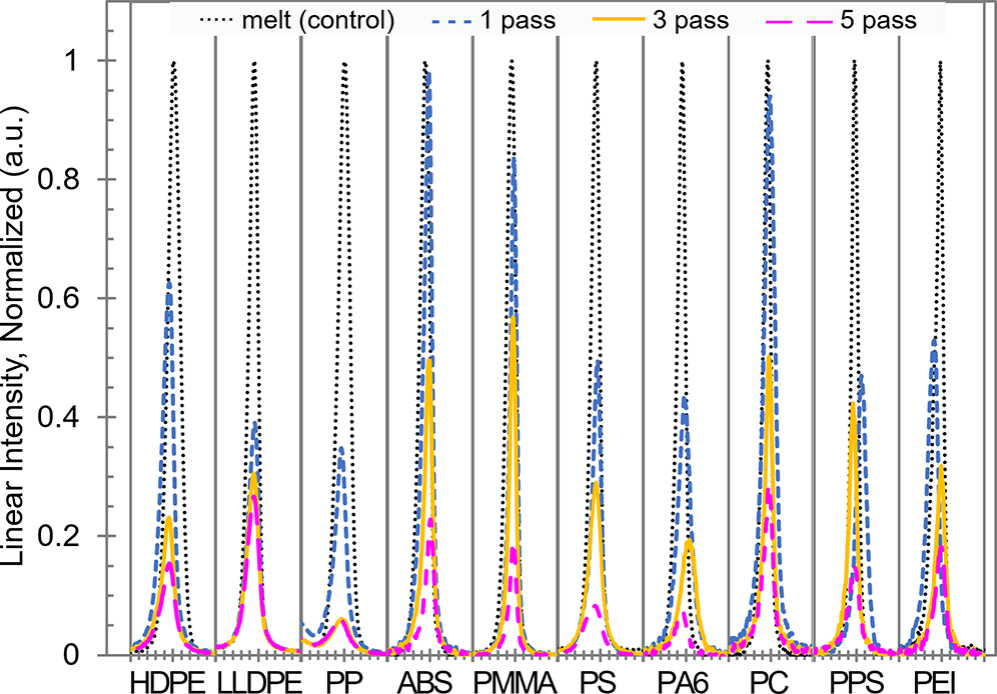
Normalized XRD spectra of polymer/2 vol % graphite materials at the characteristic graphite peak region (2θ = 26.6°). The *x*-axis range for each polymer series is 2θ = 25.6–27.6°.

In polymer nanocomposites, exfoliation of a nonisometric filler into delaminated entities is challenging but often desired to achieve physical property enhancements.^38,43^ In the case of graphite nanocomposites, nanoplatelets with a thickness of several to ∼10 nm can be exfoliated and dispersed in the polymer matrix, if rigorous compounding methods are applied.^14,42^ We employed XRD to compare the degree of graphite nanoplatelet exfoliation within and across the 10 polymer systems. The characteristic peak location of 2θ = 26.6° corresponds to an intergraphene layer spacing of 0.335 nm.^44^ An increase in the graphite exfoliation level is associated with a reduction of the characteristic peak intensity. Since the diffraction form factors are different among the 10 polymers, the X-ray spectra of the nanocomposite samples were normalized by first background-subtracting the corresponding neat polymer diffractogram and subsequently plotting the characteristic peaks in reference to the melt-processed, control polymer/2 vol % graphite sample of each polymer series. Figure 7 plots the normalized XRD spectra of 1-pass, 3-pass, and 5-pass polymer/graphite nanocomposite samples. A lower graphite peak height in Figure 7 corresponds to a more exfoliated and effectively developed nanofiller structure.

Conventional melt mixing of polymers with unmodified natural graphite without any compatibilizers results in little to no graphite exfoliation. In contrast, solid-state mechanical shearing and compression of polymeric materials with graphite particles in SSSP can yield varying degrees of an exfoliated graphite structure. Conducting a broad comparison of the 1-pass peaks, three polymers stand out with peak heights remaining tall above 0.8. ABS, PMMA, and PC were therefore particularly ineffective at exfoliating the graphite during the first pass. We explain the result with a recurring argument that these brittle polymers pulverized prematurely into fine powder at the outset of SSSP, and had limited shearing and compounding interaction with graphite downstream of the barrels.

A majority of the base polymers exhibited visible reduction in the characteristic graphite peak in their 1-pass samples, though to varying degrees. Regardless of their macroscale visual appearances in Figure 6, these thermoplastics had sufficient contact time and toughness to mechanically shear into graphite particles to exfoliate them when compounded in the solid state. In the case of PS, the earlier assertion that the partial devitrification caused thorough mixing and distribution of the graphite particles can be extended to the exfoliation phenomenon, according to Figure 7. This result highlights an intriguing point that a polymer that is typically considered nonengaging with SSSP, because of its high *T*_g_ and/or ambient brittle nature, can be facilitated to respond to SSSP compounding better when the processing temperature is near its *T*_g_ and the material is partially devitrified.

Regardless of the 1-pass spectrum graphite peak height, all polymer systems exhibit progressively lower peak height with 3 and 5 passes. Subsequent SSSP processing produces more exfoliated graphite nanoplatelets in each composite system. In the case of PP and PA6, the graphite peak reduced to <10% of the respective control peak, indicative of a significant exfoliation of the fillers. Such favorable morphology is expected to significantly improve the physical properties and practical performance of the nanocomposite.

#### 4.2.3 Property Enhancements

Lastly, we conducted property and functional performance characterizations that would confirm and directly reflect the graphite nanoplatelet dispersion levels observed above. Several different physical properties were considered for this metric, from mechanical to electrical and thermal conductivity.^14,17,39,40^ Upon extensive preliminary evaluations, we moved forward with two characterization methods that allowed all 10 polymer bases to be compared as most unbiasedly and equitably as possible. Melt rheology is one such method, where dispersed graphite nanofiller entities cause the system’s dynamic flow to transition to a solid-like behavior;^17,39,40^ shear storage and loss moduli increase and deviate from the viscous flow regime and the complex viscosity increases significantly at a lower angular frequency range.^35^ An established metric to quantify the level of nanofiller dispersion is called the shear-thinning exponent (STE),^45−47^ which is the power *n* in the following power law expression that describes the low-frequency portion of dynamic frequency sweep data.
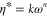
2In eq 2, η* is the dynamic viscosity, *k* is a sample-specific preexponential factor, and ω is the oscillation frequency. In practice, *n* is the slope of the straight-line segment fitted by plotting log η* vs log ω.

The STE values calculated for neat and nanofilled samples across 10 polymers, summarized in Table 2, span a wide range and do not immediately lead to clear trends or interpretations. After all, STE depends on many factors including the filler size/surface area, base polymer viscosity, and measurement temperature, which we acknowledge are not consistent in this investigation. We therefore refrain from drawing major conclusions from the overall STE comparison. However, on a basic first-order level, one can observe that most polymers exhibited a positive change in STE upon nanofiller incorporation. In particular, PP, PS, PA6, and PPS recorded a large step change between unfilled and nanofilled samples and further a monotonic increase with SSSP process passes. These four polymers with prominent shear-thinning behavior are the same four polymers displaying high levels of graphite nanoplatelet exfoliation in Figure 7. On the other hand, PMMA, PC, and PEI nanocomposites resulted in a monotonically decreasing STE with the number of passes, which suggests that the graphite nanoplatelets are not effectively involved in providing the expected rheological property enhancement. A few of the same polymers also displayed a lack of nanoplatelet exfoliation in the XRD analysis above. Thus, our results confirm that nanofiller exfoliation and dispersion directly influence the resulting rheological properties.

**Figure 8:**
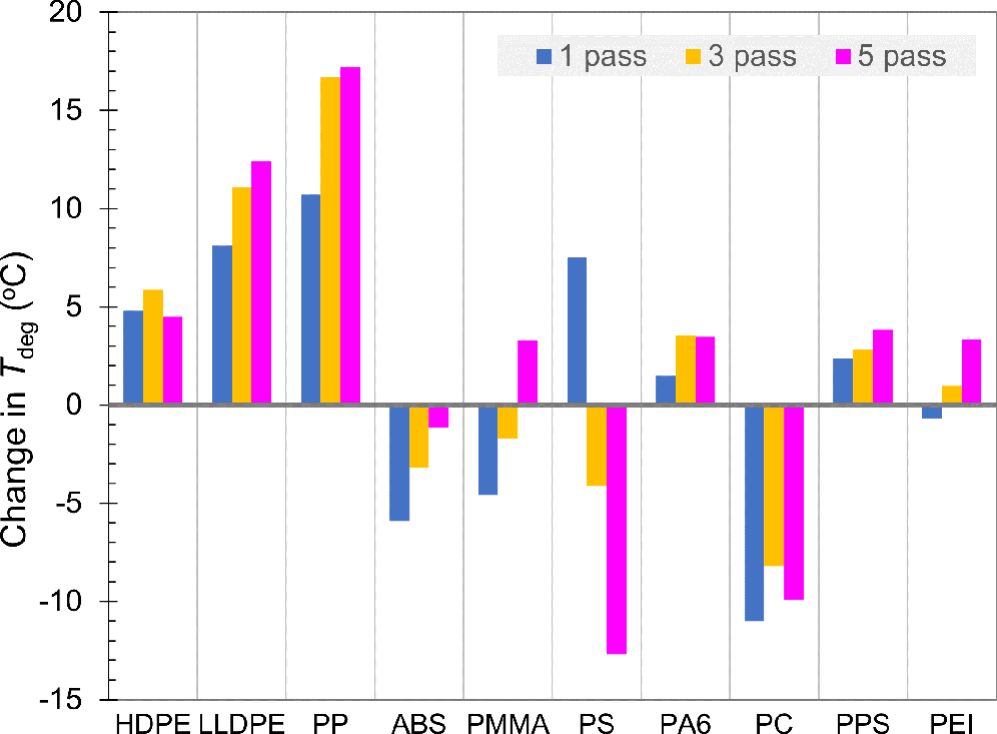
Changes in *T*_deg_ with respect to the respective neat polymer for the series of polymer/2 vol % graphite nanocomposites compounded by SSSP with 1, 3, and 5 passes.

The strongly bonded carbon-based graphite and graphite-derived nanofillers often improve the thermal stability of polymers by acting as thermal/transport barriers in the polymer matrix.^16,17,40^ We employ *T*_deg_, defined as the temperature at 5 wt % loss from the TGA results, as a metric for the degree of graphite nanoplatelet exfoliation;^40,48,49^Figure 8 displays the changes in *T*_deg_, in reference to the respective neat polymer, for the 1-, 3-, and 5-pass trials of the 10 polymer/2 vol % graphite systems.

We analyze the wide-ranging results by categorizing them into three groups. First, polyolefins exhibited effective thermal stability enhancements, including >15 °C increases observed in the PP/graphite series. These enhancements are consistent with previous reports and reinforce the notion that PP is highly compatible with graphite-based fillers.^14,17,42^ Second, some polymers, such as ABS, PMMA, and PC, exhibited negative effects in thermal stability with graphite incorporation. This is rather surprising because the XRD results above indicate that appreciative levels of graphite nanoplatelet exfoliation occurred in most samples. The downturn of their thermal stability can be attributed to the lack of physical and interfacial contact between the exfoliated fillers and the host polymer matrix. As discussed earlier, the graphite particles generated from the compounding with brittle polymers was not fully incorporated or encapsulated in the polymer flakes and particles. Instead, the changes in their thermal stability are more heavily influenced by the negative impacts from the modest chain scission phenomena in these polymers.

Lastly, PS/graphite is the only series in which *T*_deg_ progressively decreases with additional SSSP passes. Although the 1-pass nanocomposite had a positive enhancement in thermal stability, drastic declines of *T*_deg_ in 3 and 5 passes reflect the rate at which PS chains significantly degraded with additional SSSP processing. This result is consistent with the excessive reductions in effective *M*_w_ observed in PS earlier. The SSSP processing of PS near its devitrification point can lead to positive phenomena such as enhanced dispersion and distribution of filler materials, which is counteracted by substantial degradation of the matrix polymer chains. In practice, the multitude of SSSP-processing parameters may be tuned and controlled to strike an appropriate balance of two opposing sets of mechanochemical effects.

## 5 Conclusions

For the first time in 30 years of SSSP technology, systematic processing–structure–property relationships of 10 thermoplastic polymer systems were investigated, both in a neat form and with a model filler material. The wide range of mechanochemical response and structure and property changes observed across the series confirms that SSSP is by no means a universal process with some common optimal conditions. While our observations from using typical SSSP conditions highlighted that tough, semicrystalline polymers possessing high heat capacity and thermal diffusivity are most suitable in engaging with the chilled SSSP screws, it was also found that SSSP parameters, particularly those related to processing temperature, can have a significant impact on the resulting structure and properties.

No single material characteristic predicts a priori how a polymer interacts and engages with SSSP. It is important to recognize that processing conditions must be tailored to the desired shear and compression level for a given polymer system and for a specific property enhancement. After all, a unique strength of SSSP is its robust tunability in a multifaceted parameter space while still being a continuous, industry-applicable process. A future exploration area is parallel construction of low and high barrel temperature profiles, and intentional screw configuration tailored to particular processing or material performance needs.^22,25,50^

## Supporting Information Available

Zero-shear viscosity data from the neat polymer processing study (PDF)
