# Supplementary material for: Effects of Polymer Properties on Solid-State Shear Pulverization: Thermoplastic Processability and Nanofiller Dispersibility
Source: ACS Appl Polym Mater. 2023 Feb 9;5(3):1848–58. doi: 10.1021/acsapm.2c01932 (PMC10034747; doi:10.1021/acsapm.2c01932)
Supplement: Supplementary file 1 — ap2c01932_si_001.pdf [file ap2c01932_si_001.pdf]

Supporting Information

Effects of Polymer Properties on Solid-State Shear Pulverization: Thermoplastic Processability and Nanofiller Dispersibility

Tyler A. Will[1], Yiran Lu[1], and Katsuyuki Wakabayashi[1]*

[1]Department of Chemical Engineering, Bucknell University, Lewisburg, PA 17837-2029, USA

*Corresponding author: Department of Chemical Engineering, Bucknell University, 1 Dent Drive, Lewisburg, PA 17837, USA. Tel.: +1 570 577 3778; Fax: +1 570 577 1141; E-mail: kw025@bucknell.edu

TABLE S1. Comparison of the 10 Polymers in the Neat Polymer Processing Study: Zero-Shear Viscosity and Change in Effective Molecular Weight before vs after SSSP

**Polymer**	T (°C)	$\eta_{0,\,initial}$ **(Pa.s)**	$\eta_{0,\,post\text{-}SSSP}$ **(Pa.s)**	$\Delta(\eta_0^{\frac{1}{3.4}})$ **(%)**
HDPE	200	67×10^3	63×10^3	- 2
LLDPE	200	3.6×10^3	3.3×10^3	- 3
PP	210	10×10^3	7.1×10^3	- 10
ABS	230	150×10^3	170×10^3	+ 4
PMMA	230	10×10^3	7.8×10^3	- 7
PS	200	21×10^3	3.0×10^3	- 44
PA6	240	0.81×10^3	0.31×10^3	- 25
PC	220	9.2×10^3	11×10^3	+ 6
PPS	300	0.30×10^3	0.24×10^3	- 6
PEI	290	64×10^3	61×10^3	- 1
